# Battling Phages: How Bacteria Defend against Viral Attack

**DOI:** 10.1371/journal.ppat.1004847

**Published:** 2015-06-11

**Authors:** Kimberley D. Seed

**Affiliations:** Department of Molecular, Cellular, and Developmental Biology, University of Michigan, Ann Arbor, Michigan, United States of America; University of North Carolina at Chapel Hill School of Medicine, UNITED STATES

## Introduction

**Fig 1:**
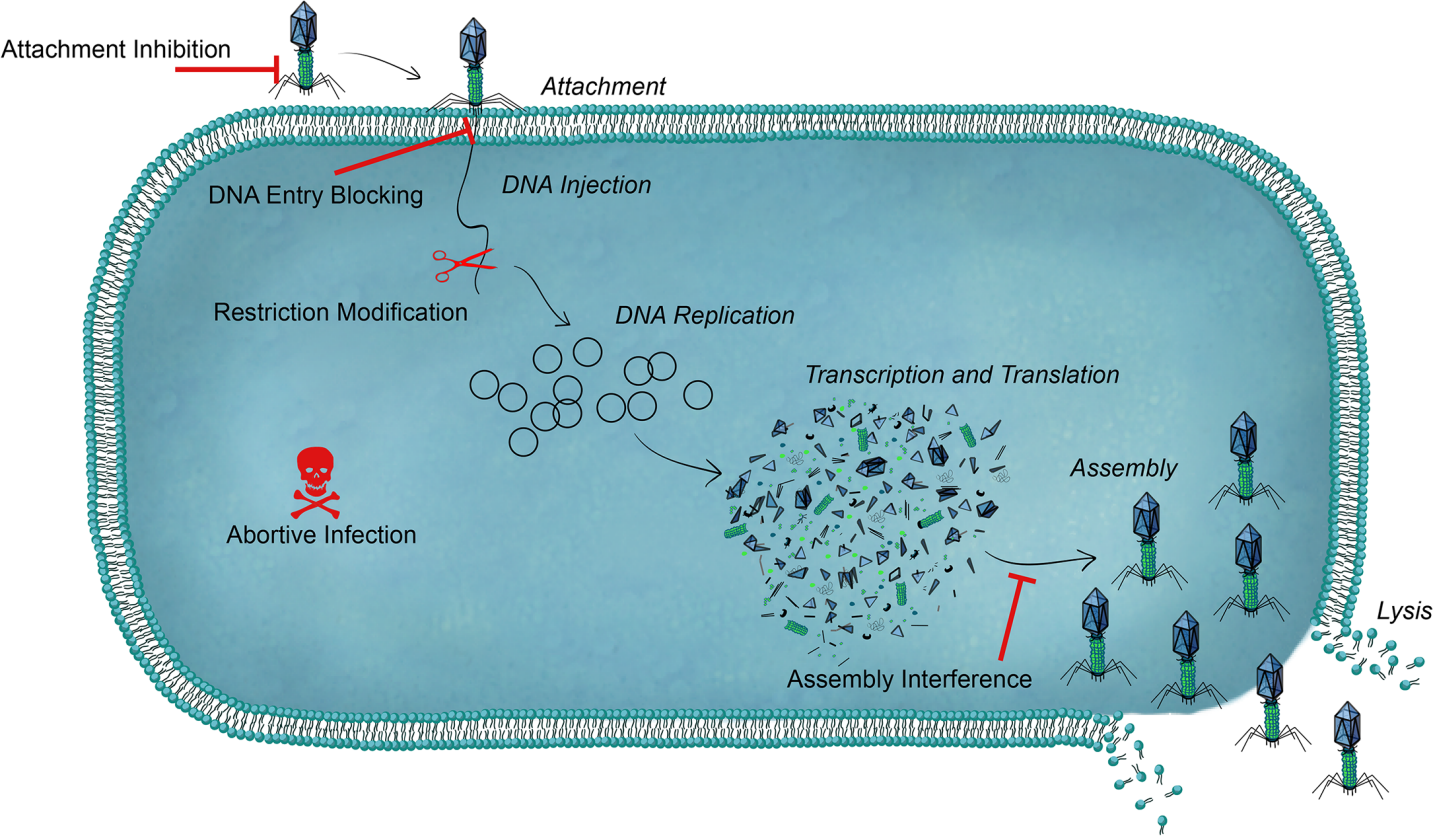
An overview of bacterial defense systems against phage. Each step of the phage lytic replication cycle is shown in italics. For simplicity, the cell wall and outer membrane (for gram-negative bacteria) is not shown. Bacteria can use a range of antiphage systems that can target all stages of the phage life cycle.

Bacteriophages (phages) are accomplished, bacteria-specific, viral predators with far-reaching impact: from the food and biotechnology industries [1] to global nutrient cycling [2] to human health and disease [3]; wherever bacteria thrive, it seems, so do predatory phages. In order to survive the constant onslaught of phage, bacteria have evolved mechanistically diverse defense strategies that act at every stage of the phage life cycle (Fig 1) [4,5]. Phages rapidly co-evolve to overcome these barriers, resulting in a constant, and often surprising, molecular arms race [6]. In this review, I highlight the spectrum of “innate” strategies used by bacteria to evade phage predation, with particular attention paid to more recent findings in the field. For a discussion of the CRISPR-Cas adaptive immune system, readers are directed to several recent reviews [4–6].

## Preventing Phage Attachment

A successful phage infection starts with adsorption of the virus to a specific bacterial surface receptor. Phage receptors, typically protein, polysaccharide, or lipopolysaccharide (LPS), must not only be present on the surface of the cell, but must be accessible and in a permissive spatial distribution. Therefore, strategies to prevent phage adsorption include modifying receptor structure through mutation and concealing receptors with an additional physical barrier [4,5]. A decrease in receptor availability can be mediated by phase variation in which receptor expression is subject to heritable, reversible switching, allowing for population heterogeneity in an effort to ensure survival. *Bordetella bronchiseptica* varies between the Bvg^+^ phase, which is required for pulmonary colonization, and the Bvg^-^ phase. In the Bvg^+^ phase, the bacteria express numerous virulence and colonization factors, including the adhesin pertactin. Phages that use pertactin as a receptor have been identified [7]. Not surprisingly, these are temperate phages associated with clinical isolates of *B*. *bronchiseptica*. Similarly, *Vibrio cholerae* O1 serogroup strains rely on expression of the LPS O1 antigen for efficient colonization of the intestinal tract. Prevalent phages associated with *V*. *cholerae* in clinical samples, although in a virulent, not temperate, relationship, depend on wild-type levels of O1 antigen expression. The O1 antigen is subject to phase variation, and these phase variants are protected from phage infection and attenuated for virulence [8]. Although the classical view of phase variation is often that its purpose is to facilitate evasion of the host immune system [9], phages also apply powerful selective forces on bacterial surface molecules, and the high levels of variation observed with many of these molecules may be driven by either, or both, forces. The expression of surface receptors can also be modulated by competing phages. The availability of the *Pseudomonas aeruginosa* type IV pilus (TFP), which is important in pathogenesis and biofilm formation, can be modulated by lysogenic conversion. Phage D3112 encodes a protein called Tip that binds to a TFP ATPase and prevents its localization, resulting in a loss of surface piliation and protection from other phages that depend on TFP for infection [10].

In some cases, phage receptors may be hidden behind a physical barrier, such as a capsule or other extracellular polymer. The K1 capsule of *Escherichia coli* has been shown to directly interfere with phage T7 attachment to its LPS receptor [11]. In addition to hiding receptors to prevent phage attachment, bacteria may produce decoys. Phage T4 levels can be reduced by the presence of outer membrane vesicles (OMVs), leading to the suggestion that shedding of OMVs into the environment may act as a decoy to prevent phage adsorption that would otherwise lead to a productive infection [12].

## Blocking DNA Entry

Following attachment to a suitable surface receptor, superinfection exclusion (Sie) systems can act to block phage DNA injection into host cells. Sie systems are typically phage encoded and act to protect a lysogenized host from infection by other, often closely related, phages. The Sie systems that have been described mechanistically are membrane-anchored or membrane-associated proteins. The *Streptococcus thermophilus* phage TP-J34 produces the Ltp_TP-J34_ membrane-localized lipoprotein, which is thought to interact with the tape measure protein of other phages [13]. Since the tape measure protein in *Siphoviridae* is involved in channel formation for DNA passage, Ltp_TP-J34_ blocks the injection process and renders the incoming phage non-infectious. The *E*. *coli* phage HK97 produces gp15, a predicted transmembrane protein that inhibits DNA entry of HK97 and the closely related phage HK75 [14]. Although several injection-blocking Sie systems have been identified, there are still many details yet to be elucidated regarding the mechanistic basis for their activity. These systems likely provide a strong selective advantage to the bacterium because, unlike the receptor blocking strategies, Sie systems conceivably protect not only the specific cell confronting phage superinfection but also the surrounding population, as the infecting phage is rendered non-infectious following DNA ejection.

## Restriction-Modification Systems

If a phage successfully adsorbs and injects its DNA into a bacterium, several lines of intracellular innate defenses may be in place to prevent phage replication and release. One such barrier is restriction-modification (R-M) systems that can destroy invading DNA. Classically, R-M systems are composed of a restriction endonuclease (REase) and a cognate methyltransferase (MTase) [15]. The MTase normally methylates self-DNA at specific recognition sites, whereas foreign DNA may be unmodified. The R-M REases recognize this unmodified DNA and cleave it into harmless fragments. R-M systems are widely distributed and rather diverse: they are classified into four types according to their subunit composition, recognition site, and mechanism of action [16]. Phages can incorporate modified bases to resist classical R-M systems [6]; however, some bacteria have modification-dependent REases (e.g., MrcBC in *E*. *coli* [17]) that act only on modified DNA.

## Abortive Infection

The phage resistance strategies described thus far all result in survival of the bacterial cell facing viral challenge. In contrast, abortive infection (Abi) systems lead to death of the infected cell as a sacrifice to protect the surrounding clonal population from predation. Abi systems are often encoded by mobile genetic elements, including prophages and plasmids [6]. These systems are mechanistically diverse and can act at any stage of phage development to decrease or eliminate the production of progeny viruses. The RexAB system in phage lambda protects lysogenized cells from infection by many other coliphages by inducing a loss of membrane potential, leading to decreased ATP levels [18]. Over 20 Abis, designated AbiA to AbiZ, have been found in *Lactococcus lactis*, a bacterium that faces phage attack during its extensive use in cheese-making fermentation processes [19]. AbiP acts early in the phage replication cycle to disrupt both phage DNA replication and the temporal switch from early to late gene expression [20]. AbiZ induces premature lysis of infected cells, ensuring that viral assembly is incomplete and infectious virions are not released [21]. Toxin-antitoxin (TA) systems have recently been shown to mediate Abi [22]. For example, the widespread AbiE system induces bacteriostasis [23] to prevent phage proliferation.

## Assembly Interference

The phage-inducible chromosomal islands (PICIs) of gram-positive bacteria are phage parasites that have the capacity to interfere with the reproduction of certain phages [24]. The best-studied members of this growing family of PICIs are the *Staphylococcus aureus* pathogenicity islands (SaPIs), which carry and disseminate critical virulence factors [25]. SaPIs reside stably in the bacterial chromosome but are induced to excise, replicate, and package themselves upon infection by specific “helper” phages. All SaPIs described thus far affect helper phage particle assembly and DNA packaging, but in contrast to other phage-resistance mechanisms, SaPIs must permit the intracellular phage program to progress so as to allow for the production of mature phage particles loaded with SaPI DNA rather than phage DNA [26]. As with the Abi systems, the infected cell dies as a consequence of phage infection, but phage reproduction is limited and SaPIs are spread to neighboring cells. SaPIs use several unique strategies to interfere with phage reproduction. They can remodel the phage capsid proteins to generate small capsids that are tailored to the smaller SaPI genome and exclude the larger helper phage genome [24,27]. SaPIs encode phage packaging interference (Ppi) proteins, which are thought to block the phage terminase small subunit (required for recognition of phage DNA and initiation of packaging), permitting the SaPI terminase small subunit to bind the phage-encoded large subunit to cleave SaPI DNA for packaging [24]. A third interference mechanism involves interrupting phage late gene activation, which is essential for phage packaging and cell lysis [26]. A PICI-like element in *V*. *cholerae* was recently shown to inhibit a virulent phage [28], although the mechanistic basis for this activity is not yet known.

## Conclusions

The strong selective pressure exerted by phages plays a key role in controlling the number and composition of bacterial populations in most, if not all, ecosystems. Conversely, bacterial strategies to resist phage attack function by controlling phage numbers and composition, thus helping to establish a predator–prey dynamic equilibrium. Many phage resistance strategies depend on the use of horizontally acquired, “selfish” elements (plasmids and prophages) that can provide efficient barriers to phage infection but that do not compromise the physiological integrity of their host cell. Thus, many of the phage resistance strategies outlined here represent competitive advances between mobile parasitic elements that depend equally on their bacterial host for long-term survival. Regardless of the origin of these systems, the consequences of the interplay between bacteria and phages necessitate molecular characterization of the many antiphage systems that are not fully understood.
